# Survival status and predictors of mortality in severely malnourished children admitted to Jimma University Specialized Hospital from 2010 to 2012, Jimma, Ethiopia: a retrospective longitudinal study

**DOI:** 10.1186/s12887-015-0398-4

**Published:** 2015-07-15

**Authors:** Habtemu Jarso, Abdulhalik Workicho, Fessahaye Alemseged

**Affiliations:** Department of Epidemiology, College of Health Science, Jimma University, P.O.Box 378, Jimma, Ethiopia

**Keywords:** Survival status, Predictors of mortality, Severe malnutrition, Children, Hospital

## Abstract

**Background:**

Although community based treatment of severe acute malnutrition has been advocated for in recent years, facility based treatment of severe acute malnutrition is still required. Therefore, information on the treatment outcomes of malnutrition and potential predictors of mortality among severely malnourished children admitted to hospitals is critical for the improvement of quality care. Thus, the aim of this study was to assess survival status and predictors of mortality in severely malnourished children admitted to Jimma University Specialized Hospital from September 11, 2010 to September 10, 2012.

**Methods:**

Retrospective longitudinal study was conducted at Jimma University Specialized Hospital. From September 11, 2010 to September 10, 2012 available data from severely malnourished children admitted to the hospital were reviewed. Data were analyzed using SPSS version 20 for windows. Bivariate and multivariable analyses were performed by Kaplan-Meier and Cox regression to identify clinical characteristics associated with mortality.

**Result:**

A total of 947 children were enrolled into the study. An improvement, death and abscond rate were 77.8, 9.3 and 12.9 % respectively. The median duration from admission to death was 7 days. The average length of stay in the hospital and average weight gain were 17.4 days and 10.4 g/kg/day respectively. The main predictors of earlier hospital deaths were age less than 24 months (AHR = 1.9, 95 % CI [1.2–2.9]), hypothermia (AHR = 3.0, 95 % CI [1.4–6.6]), impaired consciousness level (AHR = 2.6, 95 % CI [1.5–4.5]), dehydration (AHR = 2.3, 95 % CI [1.3–4.0]), palmar pallor (AHR = 2.1, 95 % CI [1.3–3.3]) and co-morbidity/complication at admission (AHR = 3.7, 95 % CI [1.9–7.2]).

**Conclusion:**

The treatment outcomes (improvement rate, death rate, average length of stay in the hospital and average weight gain) were better than most reports in the literatures and in agreement with minimum international standard set for management of severe acute malnutrition. Intervention to further reduce earlier deaths should focus on young children with hypothermia, altered mental status, dehydration, anemia and comorbidities.

## Background

Adequate nutrition is a basic human right and a pre-requisite for good health [1]. Malnutrition remains one of the most common causes of morbidity and mortality among children throughout the world and more commonly in sub-Saharan Africa and south Asia [2]. Many nutritional studies have demonstrated that malnutrition in Ethiopia is serious problem [1]. According to the Ethiopian Demographic and Health Survey (EDHS) 2011 report, stunting, under weight and wasting rates among under five year old children were 44, 29 and 10 % respectively [3].

Malnutrition is an underlying factor in over 50 % of 10–11 million children under 5 years of age who die each year of preventable causes worldwide [4]. Under-nutrition is associated with >50 % of all childhood mortality in developing countries for which infection is the underlying cause [5–8]. In Ethiopia it is estimated that malnutrition contributes to an estimated 270,000 deaths of under-five children each year [1].

Among the principal causes of death in young children, 60.7 % of deaths from diarrhea, 52.3 % of deaths from pneumonia, 44.8 % of deaths from measles, and 57.3 % of deaths from malaria are attributable to under-nutrition [8]. Because of this high risk of death, many children with severe acute malnutrition (SAM) are managed in hospitals. The number of children hospitalized with severe malnutrition continues to rise in Sub-Saharan Africa and unfortunately, many of them die [7]. In many health facilities the mortality rate from severe malnutrition at present is over 20 % [9]. Even at university hospitals case-fatality rates may be over 30 % [10]. No hospital study in sub-Saharan Africa has demonstrated a reduction of the case fatality to an acceptable international level of <5 % [7].

Although community based treatment of severe acute malnutrition has been advocated for in recent years, many children still require facility-based treatment. Unfortunately, adequate information on treatment outcomes and clinical characteristics associated with mortality that could be used to improve inpatient treatment for severe acute malnutrition is not available. Therefore, this study was conducted to assess survival status and predictors of mortality in severely malnourished children admitted to Jimma University Specialized Hospital.

## Methods

### Study design, setting, participants and data sources

A retrospective longitudinal study was conducted at Jimma University Specialized Hospital (JUSH). The hospital is the only teaching and referral hospital in the southwestern part of Ethiopia. It provides services for approximately 9000 inpatient and 80,000 outpatient attendances a year coming to the hospital from the catchment population of about 15 million people. Severely malnourished children are directly admitted to NRU and treated by Interns (Medical and Health Officer), Nurses, Residents and/or Pediatricians. NRU (sub-section of pediatrics ward of the hospital) is just one of traditional treatment centers for SAM. Admission, treatment and discharge of severe acute malnutrition were as per the Protocol for the Management of Severe Acute Malnutrition, Ethiopia – Federal Ministry of Health, March 2007 [9] which is the update of guideline for the management of severe malnutrition endorsed by the Ministry of Health in May 2004.

Study participants consist of all 947 eligible (out of total 997 SAM patients admitted to NRU from September 11, 2010 to September 10, 2012 ) severely malnourished children . The power of the study was calculated by EpiInfo for all of potential predictors identified in this study. The minimum power calculated was 83.5 % indicating adequate size of the study population was included into the study.

Admission criteria for the ward were as follows: Infants less than 6 months or less than 3 kg being breast-fed were admitted if too weak or feeble to suckle effectively (independent of weight-for-length) or if they had Weight-for-Length (W/L) less than 70 % or bilateral oedema. Children 6 months to 18 years were admitted if they had W/H or W/L < 70 % or MUAC < 110 mm with a length > 65 cm or bilateral pitting oedema. Admissions to intensive care unit (ICU) and surgical ward were excluded as these were not taken to NRU ward. Children with unknown treatment outcome and whose records were not found were also excluded from the study.

Data were collected after permission to conduct the study was obtained from the ethical clearance committee of College of Public Health and Medical Sciences, Jimma University. Permission to use the data was obtained from JUSH and department of Pediatrics, Jimma University. Confidentiality was assured by collecting data anonymously using just the card number of each record.

Data were collected by 5 BSc nurses who had experience in data collection. They also received a one day training to ensure common understanding of the data collection process. The data collection instrument was pre-tested and modified in terms of order and content. Collected data were sorted and checked for errors and completeness onsite daily by supervisors. Reviewed cards were boldly marked to avoid re-review. Data were extracted first from children’s registers and then from records (card and multi-chart). Finally, data from two sources were linked by patient’s card number. Data on variables such as patient’s card number, age, anthropometry at admission (weight, height, mid-upper arm circumference (MUAC)), length of stay in the hospital and treatment outcome were collected from SAM children’s registers. Length of stay in the hospital was also cross-checked by calculating the difference between date of admission for the current problem and date at which the patient died/lost/discharged and corrections were made where inconsistencies were found. Assessments such as vital signs and  presence or absence of clinical signs/symptoms that were made by clinicians caring for children according to hospital standards (protocol for the management of severe acute malnutrition from the Ethiopian Ministry of Health) were extracted from medical records by study nurses. Clinical conditions were evaluated and categorized according to the protocol for the management of severe acute malnutrition from the Ethiopian Ministry of Health. Co-morbidity/complication at admission was defined as co-existence of any other disease(s) with severe malnutrition at the time of admission to the hospital or manifestation of new disease(s) in addition to severe malnutrition during the first 48 h of patient’s admission to the hospital.

According to Ethiopian Protocol for the Management of Severe Acute Malnutrition, nutritional cure is when it is clear that the child is gaining weight on breast milk alone after the Supplemented Suckling technique has been used, there is no medical problem and the mother has been adequately supplemented with vitamins and minerals (for infants less than 6 months or less than 3 kg being breast-fed); when they reach 85 % weight for length and they can be switched to infant formula (for Infant less than 6 months or less than 3 kg with no prospect of being breast-fed). For children 6 months to 18 years, it is W/L ≥ 85 % or W/H ≥ 85 % on more than one occasion and absence of oedema for 10 days [9]. However, in this study, nutritional improvement, rather than nutritional cure, was considered as the endpoint because patients whose medical co-morbidities were stabilized, whose oedema disappeared and who started to gain weight were referred to nearby health facilities (health center or health post) for completion of malnutrition management. Vital signs (RR and PR) were categorized according to Advanced Paediatric Life Support [11] to enable comparison with previous literatures which have used the same classification.

### Data processing and analysis

Data were edited, entered into EpiData 3.1, exported to SPSS version 20 for Windows and cleaned to check for completeness, extreme and missing values. All statistical analyses were done using SPSS version 20 for windows. Univariate (descriptive) analyses were performed and presented by tables and graphs. Chi-square test was conducted to determine if there were adequate cell counts for each categorical variable. Kaplan-Meier and Cox regression were used to assess the association of independent variable with outcome. Before modeling, Cox regression model assumption of proportional hazards was checked by Kaplan-Meier hazard plots and testing an interaction of covariate with time. Multi-collinearity among independent variables was checked and did not found any that was significant.

During modeling, multivariable Cox regression was preceded by bivariate Cox regression. *P*-value of less than 0.2 and clinical importance were used to identify candidates for multivariable analysis. Multivariable Cox regression was run using Forward Wald method to identify best independent predictors of death. The possibilities of interactions (effect measure modification) among independent variables were explored by including interaction terms in the multivariable Cox regression. However, neither statistically significant interaction nor violation of proportional hazards assumption was found. *P*-value of less than 0.05 was considered as a statistical significance to identify independent predictors of earlier death in multivariable analysis. Hazard ratio (HR) was used as a measure of association (effect).

## Result

Out of total 997 severely malnourished children admitted to Pediatric Ward of JUSH during the study period (Sept 11, 2010–Sept 10, 2012), 947 were enrolled into the study. Fourteen (3.7 %) children with unknown treatment outcome were excluded and records for 36 eligible children registered on the register were not found.

### Socio-demographic characteristics, anthropometry and type of malnutrition

**Fig. 1:**
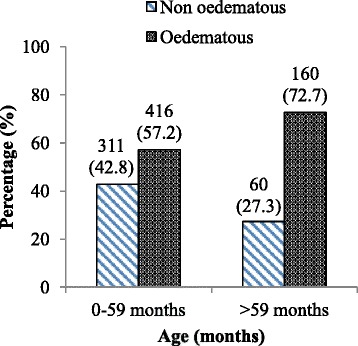
Distribution of type of malnutrition by age of severely malnourished children admitted to JUSH, Sept. 2010-Sept. 2012

More than half (58.6 %) of the children enrolled into the study were males and 68.1 % were in the age group of 6–59 months with median age of 24 months. Most (60.8 %) of the children enrolled into the study had oedematous malnutrition (kwashiorkor or marasmic-kwashiorkor). A larger proportion (42.8 versus 27.3 %) of marasmus (non oedematous malnutrition) was observed among 0–59 months old than >59 months old children whereas more oedematous malnutrition (72.7 vesus 57.2 %) was observed among >59 months old than 0–59 months old children (Fig. 1). More than half [58.5 % (87 % of non oedematous and 43 % of oedematous)] of 6–59 months old children had MUAC less than 11.5 cm; the cutoff point for severe acute malnutrition.

### Clinical profile

Most of children were in critical condition at the time admission. Out of total 947 children, 1.8 % were hypothermic (axillary temperature ≤ 35 °C), 69.2 % had deranged respiratory rate and 21.8 % had deranged pulse rate. Pale conjunctiva and palmar pallor were present in 23.6 and 18 % of the children respectively. Dehydration was present in 11.8 % of the children of which 67.9 % were severely dehydrated. Shock was present in 6.4 % of the children. Children with impaired level of consciousness (lethargic or comatose) account for 12.5 % of the total. The majority (66.0 %) and more than half (51.6 %) of the children had diarrhea and vomiting respectively where 88.8 % had watery diarrhea. Ninety eight (15.7 %) patients with diarrhea were dehydrated (of which 66 (67.3 %) were severely dehydrated). Skin lesions were present in 31.3 % of the children (Table 1).

**Table 1:** Demographic/medical features at admission stratified by treatment outcome of severely malnourished children admitted to JUSH, Sept. 2010-Sept. 2012

	Treatment outcome			
Admission Characteristics	Discharged with Improvement	Died	Absconded	Total
Socio-demographic characteristics				
Age				
<24 months	269	54	57	407
≥24 months	441	34	65	540
Sex				
Male	427	53	75	555
Female	310	35	47	392
Anthropometry and type of malnutrition				
MUAC (for 6–59 months)				
<11.5 cm	296	60	51	407
≥11.5 cm	252	8	29	289
Type of malnutrition				
Oedematous	466	46	64	576
Non oedematous	271	42	58	371
Clinical conditions at admissions				
Vital signs				
a) Hypothermia (axillary T^o^ ≤ 35 °C)				
Present	10	7	0	17
Absent	727	81	122	930
b) Respiratory rate^a^				
Bradypnea	64	10	11	85
Tachypnea	444	53	73	570
Normal	229	25	38	292
c) Pulse rate^a^				
Bradycardia	78	22	13	113
Tachycardia	73	5	16	94
Normal	586	61	93	740
Conjunctival color				
Pale	171	27	26	224
Pink	566	61	96	723
Palmar pallor				
Present	120	27	23	170
Absent	617	61	99	777
Dehydration				
Present	64	26	22	112
Absent	673	62	100	835
Shock				
Present	31	17	13	61
Absent	706	71	109	886
Consciousness level				
Impaired	72	29	17	118
Conscious^b^	665	59	105	829
Diarrhea				
Present	492	59	74	625
Absent	245	29	48	322
Vomiting				
Present	366	57	66	489
Absent	371	31	56	458
Skin lesion				
Present	226	33	37	296
Absent	511	55	85	651
Co-morbidity/complication on admission				
Co-morbidity/complication on admission				
Present	495	78	85	658
Absent	242	10	37	289
HIV Status (Unknown status = 351)				
Positive	12	7	4	23
Negative	476	38	59	573
Type of treatments given				
Infusion at admission				
Yes	36	21	19	76
No	701	67	103	871
Transfusion at admission				
Yes	3	1	1	5
No	734	87	121	942

^a^Vital sign classification – Categorical variables for respiratory rate and pulse rate were created from admission measures using cut-offs defined by levels that would imply a definite need for urgent therapeutic intervention according to Advanced Paediatric Life Support [11]

^b^ = COTPP (conscious, oriented to time, place and person), alert, irritable, apathetic

Concerning distribution of clinical conditions by type of malnutrition, the majority of children with deranged respiratory rate (55.4 %), pale conjunctiva (68.3 %), palmar pallor (68.2 %), dehydration (57.1 %) and shock (52.5 %) were those with non oedematous type of malnutrition whereas most of the children with hypothermia (64.7 %), deranged pulse rate (55.6 %), impaired consciousness level (60.2 %), acute gastroenteritis (diarrhea or vomiting) (64.0 %) and skin lesion (81.8 %) were those with oedematous type of malnutrition.

The majority (69.5 %) of the children had co-morbidity/complications on admission. More than half (54.9 %) of those with co-morbidities on admission had oedematous malnutrition. Pneumonia (32.5 %), anemia (24.6 %), disseminated TB (15.8 %) and conjunctivitis (9.9 %) were the most frequent co-morbidities/complications. Twenty-three (2.4 %) of the children were reactive for HIV test and HIV status was not known for 37 % of the children.

Of the children without co-morbidities/complications on admission, 17.0 % had developed co-morbidity/complication after admission. Pneumonia (49 %), UTI (16.3 %), acute gastroenteritis (16.3 %), oral thrush (14.3 %), conjunctivitis (10.2 %) and anemia (10.2 %) were the leading co-morbidities/complications after admission. Twenty-nine (3.3 %) of the patients without shock on admission developed shock after admission. The majority (85.7 %) of children who developed co-morbidity after admission were also those with oedematous type of malnutrition.

Characteristics at admission for the group that absconded were compared to the overall population. The groups were similar in most admission characteristics; however, there was a statistically significant (*p* = 0.006) difference in terms of resuscitation (infusion) at admission (Table 2). Only 8 % of overall population were resuscitated with IV fluid at admission, whereas twice as many (15.6 %) were resuscitated from absconded group.

**Table 2:** Comparison of characteristics at admission for the group that absconded and the overall population of severely malnourished children admitted to JUSH, Sept. 2010-Sept. 2012

Admission Characteristics	Population category			
	Overall population	Absconded	Total	*P*-value
Socio-demographic characteristics				
Age				
<24 months	407	57	464	.432
≥24 months	540	65	605	
Sex				
Male	555	75	630	.544
Female	392	47	439	
Anthropometry and type of malnutrition				
MUAC (for 6–59 months)				
<11.5 cm	407	51	458	.364
≥11.5 cm	289	29	318	
Type of malnutrition				
Oedematous	576	64	640	.076
Non oedematous	371	58	429	
Clinical conditions at admissions				
Vital signs				
a) Hypothermia (axillary T^o^ ≤ 35 °C)				
Present	17	0	17	.244^c^
Absent	930	122	1052	
b) Respiratory rate^a^				
Bradypnea	85	11	96	
Tachypnea	570	73	643	.997
Normal	292	38	330	
c) Pulse rate^a^				
Bradycardia	113	13	126	
Tachycardia	94	16	110	.530
Normal	740	93	833	
Conjunctival color				
Pale	224	26	250	.565
Pink	723	96	819	
Palmar pallor				
Present	170	23	193	.808
Absent	777	99	876	
Dehydration				
Present	112	22	134	.051
Absent	835	100	935	
Shock				
Present	61	13	74	.084
Absent	886	109	995	
Consciousness level				
Impaired	118	17	135	.645
Conscious^b^	829	105	934	
Diarrhea				
Present	625	74	699	.243
Absent	322	48	370	
Vomiting				
Present	489	66	555	.609
Absent	458	56	514	
Skin lesion				
Present	296	37	333	.835
Absent	651	85	736	
Co-morbidity/complication on admission				
Co-morbidity/complication on admission				
Present	658	85	743	.966
Absent	289	37	326	
HIV Status (Unknown status = 351)				
Positive	23	4	27	.315^c^
Negative	573	59	632	
Type of treatments given				
Infusion at admission				
Yes	76	19	95	.006
No	871	103	974	
Transfusion at admission				
Yes	5	1	6	.518^c^
No	942	121	1063	

^a^Vital sign classification – Categorical variables for respiratory rate and pulse rate were created from admission measures using cut-offs defined by levels that would imply a definite need for urgent therapeutic intervention according to Advanced Paediatric Life Support [11]

^b^ = COTPP (conscious, oriented to time, place and person), alert, irritable, apathetic

^c^Fisher exact test was used because the assumption for Pearson chi-square test was not satisfied

### Treatment outcomes

Of 947 children whose records were reviewed, 737 (77.8 %) were discharged with improvement, 88 (9.3 %) died during treatment and 122 (12.9 %) absconded (left the NRU before completing treatment). Of 88 deaths, 27.3 % occurred in the first 48 h and 60.2 % by the end of the first week. The average length of stay in the hospital was 17.4 days (16.7 for children with non oedematous and 17.9 for children with oedematous malnutrition) and the average weight gain was 10.4 g/kg/day (12.9 g/kg/day for children with non oedematous and 7.6 g/kg/day for children with oedematous malnutrition).

**Fig. 2:**
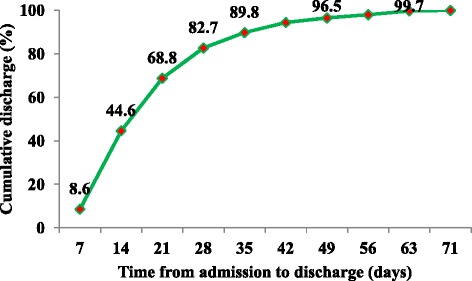
Time from admission to discharge for severely malnourished children admitted to JUSH, Sept. 2010-Sept. 2012

**Fig. 3:**
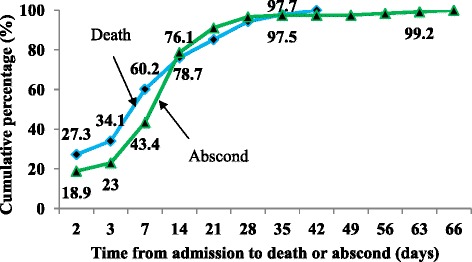
Time from admission to death or abscond for severely malnourished children admitted to JUSH, Sept.2010-Sept. 2012

Larger proportions of discharges had occurred in the second (35.8 %) and third (24.0 %) weeks of admission (Fig. 2). The mean and median duration from admission to discharge with improvement were 19.5 and 16 days respectively. Of 737 children discharged with improvement, only 226 (30.6 %) of the children achieved a target weight of 85 % weight for height. Larger proportions of deaths (60.2 %) and absconds (43.4 %) occurred in the first week of admission (Fig. 3). The mean and median duration from admission to death were 9.5 and 7 days respectively whereas mean and median duration from admission to abscond were 10.6 and 8 days respectively.

**Fig. 4:**
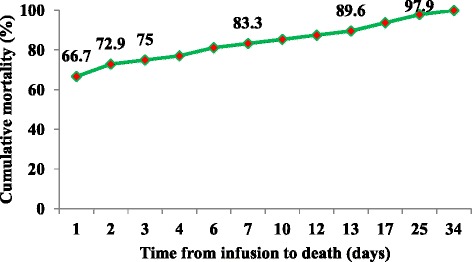
Time from infusion to death for severely malnourished children admitted to JUSH, Sept. 2010-Sept.2012

Of 947 children whose records were reviewed, 119 (12.6 %) were infused (resuscitated with IV fluid) and 14 (1.5 %) were transfused. The majority of (66.7 %) deaths among infused children occurred within first day of infusion (Fig. 4). The mean and median duration from infusion to death were 4.1 and 1 days respectively while from transfusion to death were 1.4 and 2 days respectively.

### Factors associated with earlier death of severely malnourished children

#### Bivariate analysis:

Bivariate analysis was performed for the following independent variables using Kaplan-Meier and Cox regression: socio-demographic characteristics, anthropometry and type of malnutrition, underlying clinical conditions, co-morbidity/complication at admission and type of treatments given. During regression, absconded patients and those discharged with improvement were treated as censored. In bivariate analysis, a significant difference was observed between categories for age, MUAC, hypothermia, pulse rate, palmar pallor, dehydration, shock, consciousness level, vomiting, co-morbidity/complication at admission, HIV status and infusion (Table 3).

**Table 3:** Bivariate analysis (Cox regression) of factors associated with earlier death in severely malnourished children admitted to JUSH, Sept. 2010-Sept. 2012

Factors (variables)	No	Mean Survival Time (days)	HR	95 % CI	*P*-value
Socio-demographic characteristics					
Age					
<24 months	407	49.8	2.3	1.5–3.5	< .001
≥24 months	540	66.1	1		
Sex					
Male	555	61.4	1.1	0.7–1.6	.794
Female	392	59.6	1		
Anthropometry and type of malnutrition					
MUAC (for 6–59 months)					
<11.5 cm	407	58.3	5.3	2.5–11.0	< .001
≥11.5 cm	289	60.1	1		
Type of malnutrition					
Oedematous	576	62.4	0.7	0.4–1.0	.060
Non oedematous	371	57.0	1		
Clinical conditions at admissions					
Vital signs					
a) Hypothermia (axillary T^o^ ≤ 35 °C)					
Present	17	33.4	3.9	1.8–8.4	.001
Absent	930	62.1	1		
b) Respiratory rate					
Bradypnea	85	46.1	1.4	0.7–2.9	.354
Tachypnea	570	62.2	1.0	0.6–1.6	.931
Normal	292	58.7	1		
c) Pulse rate					
Bradycardia	113	41.0	2.6	1.6–4.2	< .001
Tachycardia	94	56.9	0.6	0.2–1.5	.284
Normal	740	61.7	1		
Conjunctival color					
Pale	224	52.9	1.4	0.9–2.1	.189
Pink	723	60.6	1		
Palmar pallor					
Present	170	49.6	1.9	1.2–3.0	.006
Absent	777	62.0	1		
Dehydration					
Present	112	45.1	3.5	2.2–5.5	< .001
Absent	835	63.0	1		
Shock					
Present	61	36.5	3.7	2.2–6.4	< .001
Absent	886	62.6	1		
Consciousness level					
Impaired	118	42.9	3.8	2.4–5.9	< .001
Conscious	829	63.6	1		
Diarrhea					
Present	625	55.4	1.1	0.7–1.7	.654
Absent	322	60.7	1		
Vomiting					
Present	489	58.6	1.8	1.1–2.7	.011
Absent	458	62.7	1		
Skin lesion					
Present	296	59.3	1.3	0.8–2.0	.227
Absent	651	61.7	1		
Co–morbidity/complication at admission					
Admission co-morbidity/complication					
Present	658	59.3	3.2	1.7–6.3	< .001
Absent	289	62.5	1		
HIV Status (Unknown status = 351)					
Positive	23	38.6	4.3	1.9–9.7	< .001
Negative	573	64.5	1		
Type of treatments given					
Infusion at admission					
Yes	76	43.3	4.0	2.4–6.5	< .001
No	871	62.7	1		
Transfusion at admission					
Yes	5	36.2	2.0	0.3–14.3	.489
No	942	61.4	1		

#### Multivariable analysis:

Multivariable Cox regression was performed for variables identified by bivariate Cox regression as significant. Age less than 24 months, hypothermia (axillary T^o^ ≤ 35 °C), impaired consciousness level, palmar pallor, dehydration and co-morbidity/complication at admission were found to be independent predictors of earlier death in severely malnourished children admitted to the hospital. However, type of malnutrition, pulse rate, conjunctival color, shock, vomiting and infusion were not independent predictors of earlier death (Table 4).

**Table 4:** Multivariable analysis of factors associated with earlier death in severely malnourished children admitted to JUSH, Sept. 2010–Sept. 2012

Factors (variables)	CHR	95%CI	AHR	95 % CI	*P*-value
Socio-demographic characteristics					
Age					
<24 months	2.3	1.5–3.5	1.9	1.2–2.9	.006
≥24 months	1		1		
Clinical conditions at admissions					
Hypothermia (axillary T^o^ ≤ 35 °C)					
Present	3.9	1.8–8.4	3.0	1.4–6.6	.005
Absent	1		1		
Consciousness level					
Impaired	3.8	2.4–5.9	2.6	1.5–4.5	< .001
Conscious	1		1		
Palmar pallor					
Present	1.9	1.2–3.0	2.1	1.3–3.3	.003
Absent	1		1		
Dehydration					
Present	3.5	2.2–5.5	2.3	1.3–4.0	.004
Absent	1		1		
Co–morbidity/complication at admission					
Present	3.2	1.7–6.3	3.7	1.9–7.2	< .001
Absent	1		1		

Adjusting for other variables, children with age less than 24 months were 1.9 (95 % CI [1.2–2.9]; *p* = 0.006) times more likely to die earlier than children with age 24 and above months. Risk of earlier death for hypothermic children was 3.0 (95 % CI [1.4–6.6]; *p* = 0.005) times higher than for children without hypothermia. Children with impaired consciousness (lethargy or coma) were 2.6 (95 % CI [1.5–4.5]; p < .001) times more likely to die earlier than conscious children. Risk of earlier death for children with palmar pallor was 2.1 (95 % CI [1.3–3.3]; *p* = 0.003) times higher than children without palmar pallor. Dehydrated children were found to be 2.3 (95 % CI [1.3–3.9]; *p* = 0.004) times more likely to die earlier than children who were not dehydrated. Children with co-morbidity/complication at admission were 3.7 (95 % CI [1.9–7.2]; p < 0.001) times more likely to die earlier than children without co-morbidity/complication at admission. Treatment related factors such as infusion and transfusion were not independent predictors of death in severely malnourished children admitted to JUSH.

## Discussion

This study was conducted on 947 severely malnourished children admitted to Jimma University Specialized Hospital (JUSH) from September 2010 to September 2012 to assess survival status and identify predictors of mortality. Similar to the reports from previous studies in Ethiopia [5, 12], the majority (68.1 %) of admitted children were 6–59 months of age.

Most (60.8 %) of the children enrolled into the study had oedematous malnutrition (kwashiorkor or marasmic-kwashiorkor) similar to some previous reports [5, 6, 13]. Similar to one study in Ethiopia [14], a larger proportion (42.8 versus 27.3 %) of marasmus (non oedematous malnutrition) was observed among 0–59 months old as compared to those >59 months [15]. These findings are in contrast to studies conducted in Colombia and Kenya [13, 14]. This could be due to the differences in the causes of malnutrition in various parts of the world [5].

In this study, the death rate was 9.3 % (27.3 % in the first 48 h and 60.2 % by the end of the first week). This finding was consistent with the minimum international standard set for management of severe acute malnutrition of less than 10 % [5] and lower than the findings of most other studies [7, 10, 12, 14, 16–18]. This may have been the result of differences in patient load, patient profile, management protocol, management team and medical supplies. However, the death rate was higher than in the two studies conducted in Ethiopia [5, 6]. This could be due to the difference in treatment setup (community based where children admitted might not be as medically complicated as children admitted to JUSH) and purpose of the study (to evaluate treatment outcome of severely malnourished children treated according to UNICEF 2004 guidelines where there might be close follow up of the children with strict adherence to the guidelines).

The average length of stay in the hospital of 17.4 days (16.7 days for children with severe wasting and 17.9 days for children with oedematous malnutrition) was consistent with the minimum international standard set for management of severe acute malnutrition which is average length of stay less than 30 days [5]. However, the overall average length of stay in the hospital was longer than in other studies [6, 18]. This may have been due to the underlying medical conditions of children in our population.

The average weight gain of 10.4 g/kg/day (12.9 g/kg/day for children with severe wasting and 7.6 g/kg/day for children with oedematous malnutrition) was also in agreement with the minimum international standard set for management of severe acute malnutrition which is average weight gain of 8 g/kg/day [5]. This was far higher than in a study conducted in South Africa [18], perhaps because of differences in length of stay in the hospital or differences in study setting. However, it was lower than finding in a study conducted at a community based treatment setup [5] which could be because the stay for children at the community based treatment setup was longer.

Out of total 737 children discharged with improvement, only 226 (30.6 %) of the children had achieved target weights of 85 % weight for height at the time of discharge. This finding was lower than in a study conducted at Mulago Hospital, Uganda [7] which could be due to differences in patient load in which case children might be referred to the nearby health facility (district hospital, health center, health post and OTP centers, managing patients without complication/co-morbidity) for completion of malnutrition management after stabilization of medical co-morbidities at JUSH.

Adjusting for other variables, children with age less than 24 months were 1.9 times more likely to die earlier than children with age 24 and above months. This was in agreement with other reports [5]. Younger children may be more vulnerable because of depressed immunity, increased risk of infection and insufficient feeding practices. Children in our population with hypothermia were more likely to die than those who were not, in contrast to a study conducted in South Africa [17]. Children with impaired consciousness level (lethargy or coma) were 2.6 times more likely to die earlier than conscious children. This was similar to the finding of study conducted in Kenya [14]. Risk of earlier death for children with palmar pallor was 2.1 times higher than children without palmar pallor. This was similar to studies conducted in South Africa and Niger [17, 19]. Dehydrated children were 2.3 times more likely to die earlier than children who were not dehydrated. This might be because of misdiagnosis and mistreatment of dehydration in severely malnourished children who can quickly develop fluid overload and cardiac failure during fluid repletion [2]. Children with co-morbidities/complication at admission were 3.7 times more likely to die earlier than children without co-morbidities/complication. This could be due to increased nutrient loss and nutrient requirement in the face of decreased nutrient absorption and utilization [2].

Treatment related factors like infusion and transfusion were not independent predictors of death in severely malnourished children admitted to JUSH. This was in contrast to a study conducted in Uganda [7] where transfusion and infusion were predictors of mortality. A study conducted in South Africa [17] also found that transfusion was associated with death. This could be the result of fluid overload from inappropriate use of transfusions and infusions or from differences in study settings [2, 7].

One strength of this study is that data regarding predictors were collected at admission, before the discharge outcome was known guaranteeing that the measurement of predictor variables was not biased by knowledge of the subjects’ outcomes. However, threats to this study include potential bias associated with excluded records, unknown status of absconds and lack of control over the quality of the measurements that were made at admission. In addition, exclusion of admissions to intensive care unit (ICU) and surgical ward might have resulted in under estimation of the number of deaths. Finally, this study did not consider broad ranges of socio-demographic characteristics, biochemical findings and patient management related factors (such as medical supplies and skill of professionals) that might have influenced outcomes.

## Conclusion

Most of the severely malnourished children admitted to JUSH were not in critical condition when they came to attention. Treatment outcomes (improvement rate, death rate, average length of stay and average weight gain) of severely malnourished children admitted to JUSH were better than most reports in the literatures. The main predictors of earlier hospital deaths for severely malnourished children admitted to JUSH include age less than 24 months, hypothermia, impaired consciousness level, palmar pallor, dehydration and co-morbidity/complication at admission. However, infusion and transfusion were not found to independent predictors of death. Therefore, special attention should be paid to children with younger age, hypothermia, impaired consciousness level, palmar pallor, dehydration and co-morbidity/complication at admission in the management of severely malnourished children admitted to hospital. However, as this was a pilot study, the findings should be confirmed in another setting before widespread acceptance and utilization of the study findings occurs.

## Abbreviations

EDHS: Ethiopian Demographic and Health Survey
ENGINE: Empowering new generations to improve nutrition and economic opportunities
JUSH: Jimma University Specialized Hospital
NRUs: Nutritional rehabilitation units
OTP: Outpatient Therapeutic Program
SAM: Severe acute malnutrition
UNICEF: United Nations Children’s Fund
USAID: United States Agency for International Development
W/H: Weight for height
W/L: Weight for length
WHO: World Health Organization
